# Activation of Pro-apoptotic Caspases in Non-apoptotic Cells During Odontogenesis and Related Osteogenesis

**DOI:** 10.3389/fphys.2018.00174

**Published:** 2018-03-07

**Authors:** Eva Svandova, Barbora Vesela, Abigail S. Tucker, Eva Matalova

**Affiliations:** ^1^Department of Physiology, University of Veterinary and Pharmaceutical Sciences Brno, Brno, Czechia; ^2^Laboratory of Molecular Morphogenesis, Institute of Animal Physiology and Genetics, Czech Academy of Sciences, Brno, Czechia; ^3^Department of Craniofacial Development and Stem Cell Research, King's College London, London, United Kingdom

**Keywords:** caspase, differentiation, apoptosis, tooth, intramembranous, bone, osteocalcin

## Abstract

Caspases are well known proteases in the context of inflammation and apoptosis. Recently, novel roles of pro-apoptotic caspases have been reported, including findings related to the development of hard tissues. To further investigate these emerging functions of pro-apoptotic caspases, the *in vivo* localisation of key pro-apoptotic caspases (-3,-6,-7,-8, and -9) was assessed, concentrating on the development of two neighbouring hard tissues, cells participating in odontogenesis (represented by the first mouse molar) and intramembranous osteogenesis (mandibular/alveolar bone). The expression of the different caspases within the developing tissues was correlated with the apoptotic status of the cells, to produce a picture of whether different caspases have potentially distinct, or overlapping non-apoptotic functions. The *in vivo* investigation was additionally supported by examination of caspases in an osteoblast-like cell line *in vitro*. Caspases-3,-7, and -9 were activated in apoptotic cells of the primary enamel knot of the first molar; however, caspase-7 and -8 activation was also associated with the non-apoptotic enamel epithelium at the same stage and later with differentiating/differentiated odontoblasts and ameloblasts. In the adjacent bone, active caspases-7 and -8 were present abundantly in the prenatal period, while the appearance of caspases-3,-6, and -9 was marginal. Perinatally, caspases-3 and -7 were evident in some osteoclasts and osteoblastic cells, and caspase-8 was abundant mostly in osteoclasts. In addition, postnatal activation of caspases-7 and -8 was retained in osteocytes. The results provide a comprehensive temporo-spatial pattern of pro-apoptotic caspase activation, and demonstrate both unique and overlapping activation in non-apoptotic cells during development of the molar tooth and mandibular/alveolar bone. The importance of caspases in osteogenic pathways is highlighted by caspase inhibition in osteoblast-like cells, which led to a significant decrease in osteocalcin expression, supporting a role in hard tissue cell differentiation.

## Introduction

Tooth development proceeds via stages referred to as placode, bud, cap, and bell (Tucker and Sharpe, 2004), and is completed by differentiation of two main cell types, mesenchymal-derived odontoblasts producing dentin and epithelial-derived ameloblasts secreting enamel (e.g. Lesot et al., 2014). Odontogenesis is closely related to the development of other jaw structures including the bone (Diekwisch, 2002; Diep et al., 2009), which forms by intramembranous ossification involving direct differentiation of osteoblasts from mesenchymal precursors (Alfaqeeh et al., 2013).

Caspases are cysteine-dependent aspartate proteases (Cohen, 1997) produced as non-active zymogens (procaspases) and activated by homodimerisation or cleavage (reviewed in Boatright and Salvesen, 2003). Pro-apoptotic caspases are classified as initiators (in mouse these include caspases-2,-8, and -9) or executors (caspases-3,-6, and -7). These caspases are engaged in both major pathways of apoptosis; extrinsic (receptor mediated) and intrinsic (mitochondrial). The initiators possess a long prodomain, the prodomain of executors is short (reviewed in Nicholson, 1999). Caspases-3 and -7, although structurally/functionally related, differ in their N-terminal peptides, regions thought to be involved in subcellular targeting. Caspase-6 substrate specificity differs substantially from that of caspases-3 and -7, suggesting a non-overlapping subset of substrates (reviewed in Fuentes-Prior and Salvesen, 2004). Beyond pro-apoptotic caspases, inflammatory caspases (caspases-1, -11, and -12) have been identified in the mouse along with two additional caspases (caspases-14 and -16), which do not fit in either category (reviewed in Shalini et al., 2015). This investigation focused on the pro-apoptotic caspases with the exception of caspase-2, the position of which in the apoptotic pathway is unclear (Fava et al., 2012).

Some of the pro-apoptotic caspases have also been recently described in non-apoptotic events, such as cell proliferation, differentiation, migration, regeneration, or senescence (reviewed in Schwerk and Schulze-Osthoff, 2003; Lamkanfi et al., 2007; Connolly et al., 2014; Shalini et al., 2015), and ongoing research indicates novel, non-apoptotic functions of pro-apoptotic caspases during tooth and bone development.

Roles of pro-apoptotic caspases during tooth development have typically been classified as apoptotic, particularly in elimination of signalling centres or reduction of ameloblasts, odontoblasts, and osteogenic populations (reviewed in Matalova et al., 2012a). However, non-apoptotic appearance of caspase-7 was also detected in differentiating/differentiated odontoblasts and ameloblasts (Matalova et al., 2012b, 2013).

Related to osteogenesis, caspases have been described to regulate a number of osteogenic cells by apoptotic machinery (reviewed in Hock et al., 2001). Among non-apoptotic functions, caspases-3 and -8 were suggested to play a role in osteoblastic (Mogi and Togari, 2003) and/or osteoclastic differentiation (Szymczyk et al., 2006; Katao et al., 2013, 2014). Further, caspase-3 deficient mice show decreased total bone mineral density plus decreased osteogenic and proliferating capacity of bone marrow stromal stem cells (BMSSCs) (Miura et al., 2004). A similar tendency was earlier reported in caspase-7 deficient mice, where downregulated expression of two important osteogenic factors, *Smad1* and *Msx1*, was demonstrated (Svandova et al., 2014).

This study was designed to provide a comprehensive temporo-spatial analysis of activation of the key pro-apoptotic caspases (-3,-6,-7,-8, and -9) during development of the mouse molar tooth and surrounding bone, and to investigate non-apoptotic appearance of these caspases related to the emerging evidence about their novel roles. Additionally, the novel potential of caspases was analysed *in vitro* based on osteocalcin expression modulation in an osteoblastic cell line derived from intramembranous bone.

## Materials and methods

### Biological material

Mouse heads (ICR/CD1) at embryonic (E) stages E13, E15, E18, postnatal (P) day P0 and P22 were used for *in situ* detection of activated caspases by immunofluorescence, histological staining, TRAP, and TUNEL analysis. Collected samples were fixed in 4% buffered paraformaldehyde for at least 24 h, then dehydrated in an ethanol gradient, treated with xylene, and embedded in paraffin. Mice were sacrificed according to the experimental protocol approved by the Laboratory Animal Science Committee of the UVPS, Brno, Czech Republic.

A cell line of osteoblastic precursors, MC3T3-E1, was purchased from the European Collection of Cell Culture (c.n. 99072810) and differentiated for 21 days to allow for the differentiation of osteoblasts according to Yazid et al. (2010). Medium consisted of MEM Alpha (Gibco, USA) enriched by FCS (10%), penicillin/streptomycin (100 U/ml, 100 μg/ml), β-glycerolphosphate (10 mM), and ascorbic acid (50 μg/ml). Medium was changed every second day. Passages 12–20 were used for the experiments.

### Histological staining

Histological sections of heads were stained using trichrome staining (alcian blue, haematoxylin, and sirius red) and haematoxylin-eosin staining.

### Immunofluorescence

Histological sections were treated with xylene, rehydrated through a graded ethanol series, and pre-treated in citrate buffer (pH = 6.0) 10 min at 98°C. The primary antibodies: cleaved caspase-3 (9664, Cell Signaling, USA), cleaved caspase-6 (9761, Cell Signaling, USA), cleaved caspase-7 (9491S, Cell Signaling, USA), cleaved caspase-8 (8592, Cell Signaling, USA), cleaved caspase-9 (9509, Cell Signaling, USA), and osteocalcin (ab93876, Abcam, UK), were diluted (anti-caspase-3: 1:50, anti-caspase-6: 1:50, anti-caspase-7: 1:50, anti-caspase-8: 1:200, anti-caspase-9: 1:50, anti-osteocalcin 1:100) and applied overnight at 4°C. Alexa Fluor® 488 (A11034, Thermo Fischer, USA) or Alexa Fluor® 594 (A11037, Thermo Fischer, USA) were diluted 1:200 and then applied for 40 min at room temperature (RT). Nuclei were visualised by ProLong Gold Antifade reagent with DAPI (Thermo Fischer, USA).

### Immunocytofluorescence

MC3T3-E1 cells were cultured on histologic slides, fixed in 10% PFA, washed in PBS, and treated with 0.1% Triton X-100 for 15 min. The caspase primary antibodies and osteocalcin primary antibody were diluted as described above; all primary antibodies were applied overnight at 4°C. Alexa Fluor® 488 (A11034, Thermo Fischer, USA) was diluted 1:200 and then applied for 40 min at RT. Nuclei were visualised by ProLong Gold Antifade reagent with DAPI (Thermo Fischer, USA).

### Immunohistochemistry

Histological sections were rehydrated and pre-treated in citrate buffer as described above. Endogenous peroxidase activity was eliminated by 3% hydrogen peroxide in PBS (5 min at RT). The primary antibodies were diluted as described above and applied overnight at 4°C, peroxidase-conjugated streptavidin-biotin system (Vectastain, USA) and chromogen substrate diaminobenzidine (DAB, K3466, Dako, Denmark) reactions were used to visualise positive cells as brown. Tissues were counterstained with haematoxylin.

### Detection of osteoclasts - TRAP

Histological sections were rehydrated as described above and incubated 1 h at 37°C in a solution consisting of 0.0023 M Naphthol AS-TR phosphate disodium salt (N6125, Sigma Aldrich, Germany), 0.2 M glacial acetic acid, 0.2 M sodium acetate, 0.1 M sodium tartrate dibasic dihydrate (S-8640, Sigma Aldrich, Germany), and 0.5% N-N-dimethylformamide. Tissues were counterstained with haematoxylin.

### TUNEL assay

Slides were rehydrated as described above. Tissues were pre-treated with proteinase K 20 mg/ml (15 min at RT). Endogenous peroxidase was blocked with 3% hydrogen peroxide in PBS (5 min at RT). The reaction mixture (TUNEL, S7100, Merck Millipore, USA) was prepared as follows: 3 μl TdT enzyme, 42 μl distilled water, 105 μl reaction buffer, which was incubated for 45 min at 37°C. An anti-digoxigenin-peroxidase reaction was performed for 30 min at RT. Positive cells were finally visualised by the chromogenic substrate diaminobenzidine (DAB, K3468, Dako, Denmark). Samples were counterstained with haematoxylin.

### Caspase inhibition in MC3T3-E1 cells

General caspase inhibitor Z-VAD-FMK (FMK001, R&D Systems, Germany) was dissolved in DMSO, and applied at a final concentration of 100 μM. The medium was changed every second day. The control group was treated with the same concentration of DMSO as used for the experimental samples to exclude any side effect of this solvent. Cells were seeded at a concentration of 5000 cells/cm^2^ and harvested after 5 or 6 days of caspase inhibition. Cells were then lysed in RLT buffer and used for RNA isolation using the RNeasy Mini Kit (Qiagen, USA). First strand cDNA was transcribed using Super Script VILO (Invitrogen, USA). Inhibition of central caspase-3 at the protein level was verified using a bioluminescence approach as described earlier (Ledvina et al., 2017).

### qPCR

qPCR was performed in 10 μl final reaction volumes containing the one-step master mix gb Ideal PCR Master Mix (Generi Biotech, Czech Republic), qPCR was performed by a LightCycler 96 (Roche, Switzerland) with preheating at 95°C for 10 min, followed by 40 cycles of 95°C for 15 s and 62°C for 1 min. Osteocalcin expression levels (Mouse *Bglap*, Mm03413826_mH, TaqMan Gene Expression Assay, Thermo Fischer, USA) were calculated using the ΔΔCT method, with normalisation against actin levels (Mouse *Actb*, Mm02619580_g1, TaqMan Gene Expression Assay, Thermo Fischer, USA), which was used as the internal control. For both groups, analysis was performed in three biological replicates, reactions were performed in triplicates for each sample. Statistical analysis was performed for both groups (*t*-test, *p* ≤ 0.05).

## Results

### Development of tooth and surrounding bone

**Figure 1:**
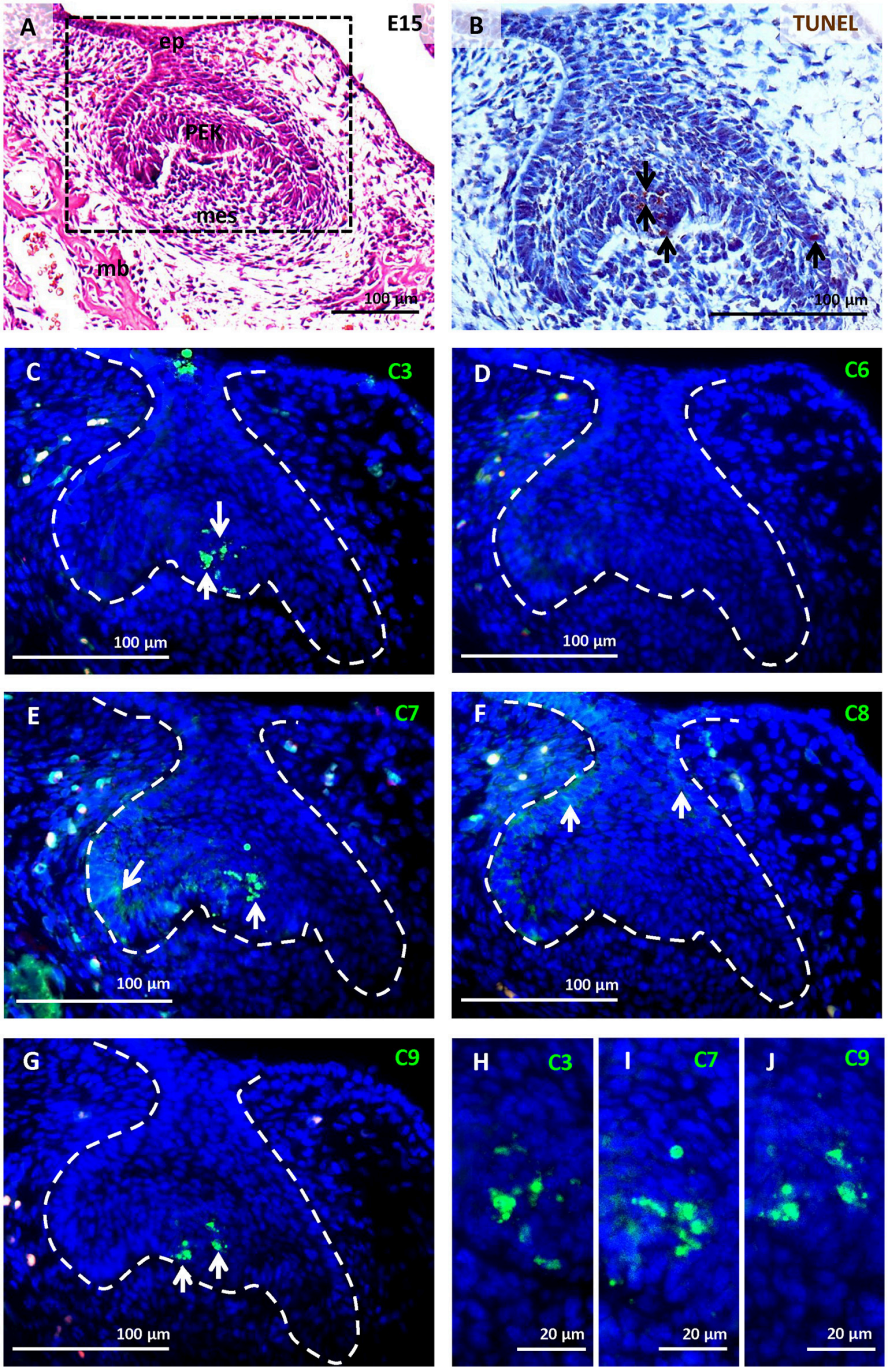
Activation of caspases in the first mandibular molar at E15. Molar morphology (early bell stage) visualised by haematoxylin-eosin staining **(A)**, apoptotic cells (brown) labelled by TUNEL assay **(B)**, immunofluorescent detection (green) of caspase-3 **(C,H)**, caspase-6 **(D)**, caspase-7 **(E,I)**, caspase-8 **(F)**, caspase-9 **(G,J)**. Nuclei were counterstained by DAPI (blue). Ep (epithelium), mb (mandibular bone), mes (mesenchyme), primary enamel knot (PEK). Arrows point to positive cells.

The mouse first lower molar is frequently used to study odontogenesis and related osteogenesis, (Cohn, 1957; Gaunt, 1966; Peterková et al., 1996); therefore, we concentrated on this region for our study. Two stages were studied for the primary comparative analysis. At E15, the first molar has reached the early bell stage and the epithelial part can be distinguished into inner and outer epithelium surrounded by the dental follicle containing undifferentiated mesenchymal cells (Figure 1A). Apoptotic elimination of the primary enamel knot (PEK) was apparent at this stage (Figure 1B; Jernvall et al., 1998). Later, at P0 (**Figure 4A**), differentiation of odontoblasts and ameloblasts proceeded and secretion of dentin was evident (**Figure 4B**).

**Figure 2:**
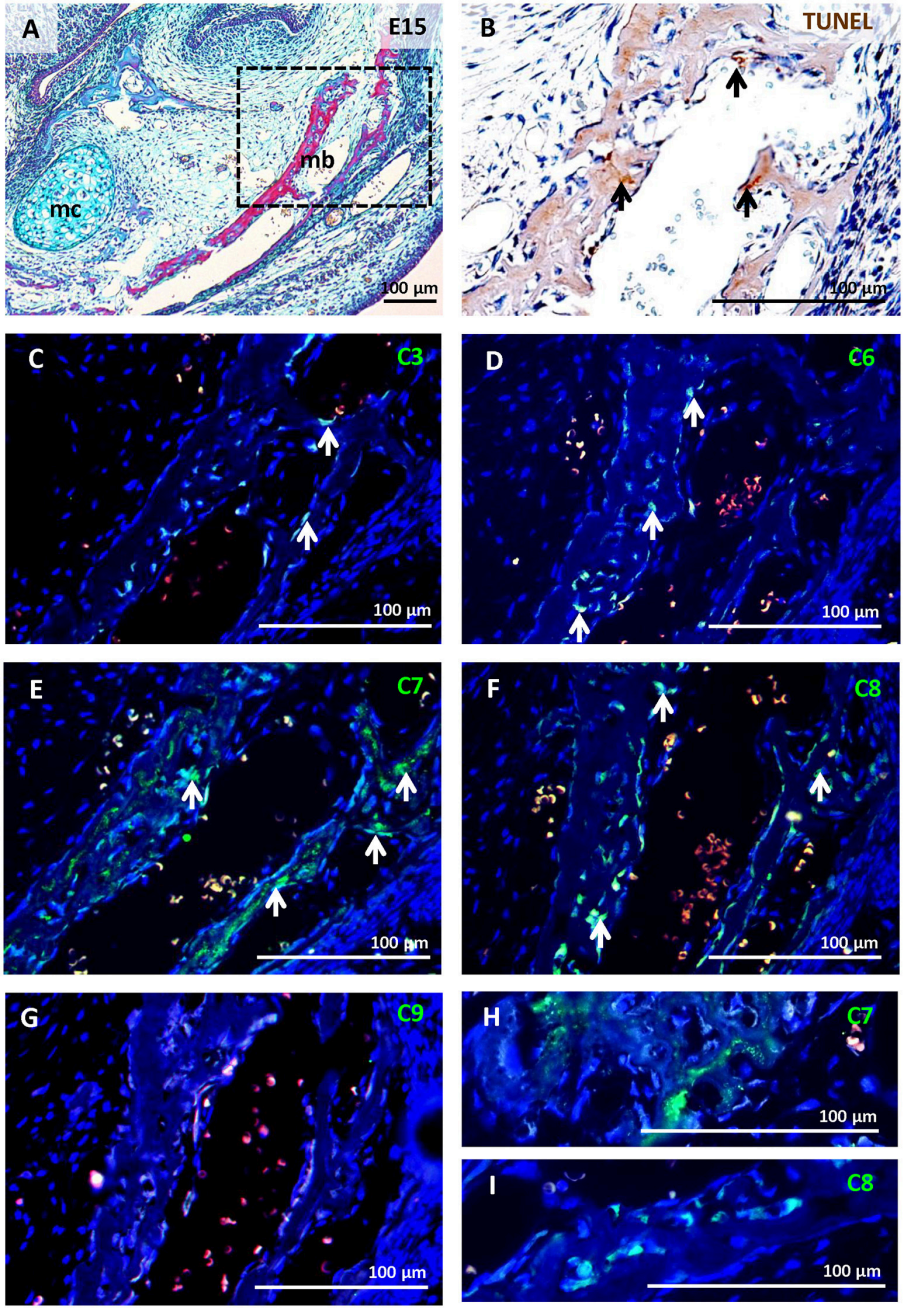
Activation of caspases in the mandibular/alveolar bone at E15. Mandibular/alveolar bone morphology visualised by trichrome staining **(A)**, apoptotic cells (brown) labelled by TUNEL assay **(B)**, immunofluorescent detection (green) of caspase-3 **(C)**, caspase-6 **(D)**, caspase-7 **(E,H)**, caspase-8 **(F,I)**, caspase-9 **(G)**. Nuclei were counterstained by DAPI (blue). Mb (mandibular bone), mc (Meckel's cartilage). Arrows point to positive cells.

**Figure 3:**
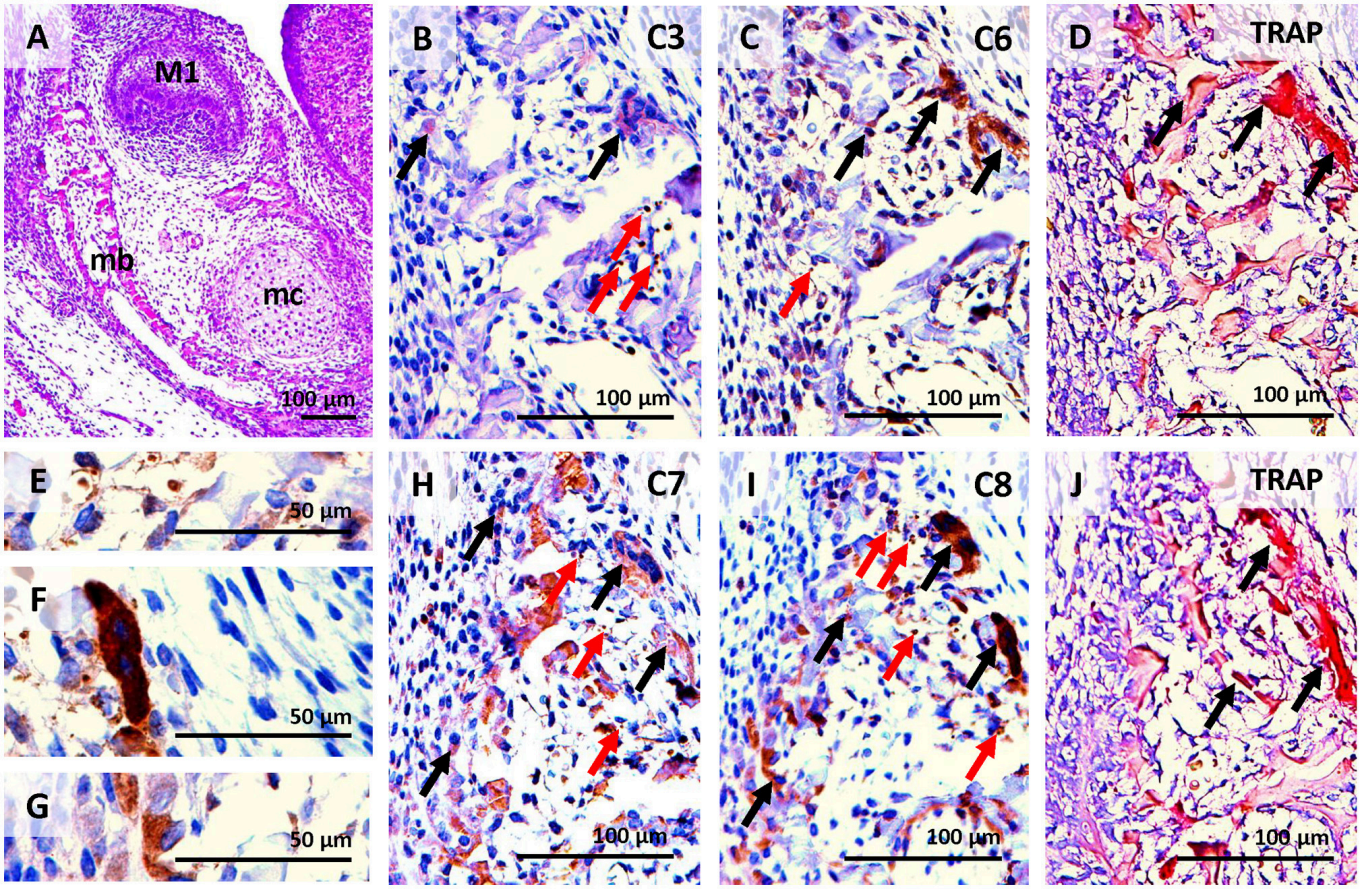
Activation of caspases correlated with osteoclast distribution detected by TRAP in serial sections of the alveolar/mandibular bone at E15. Haematoxylin-eosin staining **(A)**, activated caspase-3 **(B)**, activated caspase-6 **(C)**, osteoclast detection **(D,J)**, activation of caspase-7 **(H)**, activation of caspase-8 **(I)** localised in apoptotic bodies **(E)**, non-apoptotic osteoclast **(F)**, non-apoptotic osteoblasts **(G)**. M1 (first molar), mb (mandibular bone), mc (Meckel's cartilage). Black arrows point to intact cells, red arrows point to apoptotic cells.

Alveolar bone was already apparent at E15, surrounding the molar tooth germ and forming a functional component with the mandibular bone (Figures 2A, 3A). This stage is characterised by the first appearance of complete bone (osteoblasts, osteocytes, and osteoclasts). Activated osteoclasts appeared at this stage to start continuous dynamic remodelling of the bone (Figures 3D,J). At P0, the mandibular/alveolar bone completely surrounded the developing molar (Figure **5A**).

### Activation of caspases in the first molar tooth germ at E15

This stage of odontogenesis is characterised by high levels of apoptosis (Figure 1B) in the PEK. Activated caspase-3 (Figure 1C), caspase-7 (Figure 1E), and caspase-9 (Figure 1G) were detected and associated with the PEK region (Figures 1H–J). Activation of caspase-6 (Figure 1D) was not observed at this stage of odontogenesis. Caspase-7 and caspase-8 were both apparent in the enamel epithelium (Figures 1E,F), where apoptotic cells were not found. Activated caspases in the PEK showed nuclear localisation (Figures 1H–J), while activated caspases in other parts of the enamel organ were cytoplasmic (Figures 1E,F).

### Activation of caspases in mandibular/alveolar bone at E15

Apoptotic cells were already detected in the area of the bone at E15 (Figure 2B). Only sporadic activation of caspase-3 was apparent in osteoblasts and osteoclasts (Figures 2C, 3B). Activation of caspase-6 was present in some osteoblasts and osteoclasts, which were mostly non-apoptotic (Figures 2D, 3C). Activation of caspase-7 and -8 was abundant at this stage, it was apparent in osteoblasts as well as osteoclasts (Figures 2E,F,H,I, 3H,I). However, no caspase-9 activation was observed (Figure 2G).

Some bone cells with activated caspases-3,-7, or -8 showed apoptotic features, these apoptotic cells exhibited nuclear localisation of caspases (e.g. Figure 3E). The majority of osteoblasts (Figure 3G) and osteoclasts (Figure 3F) with activated caspases-6,-7, and -8 were not apoptotic and showed cytoplasmic localisation (Figures 3C,H,I).

### Activation of caspases in first molar development at P0

**Figure 4:**
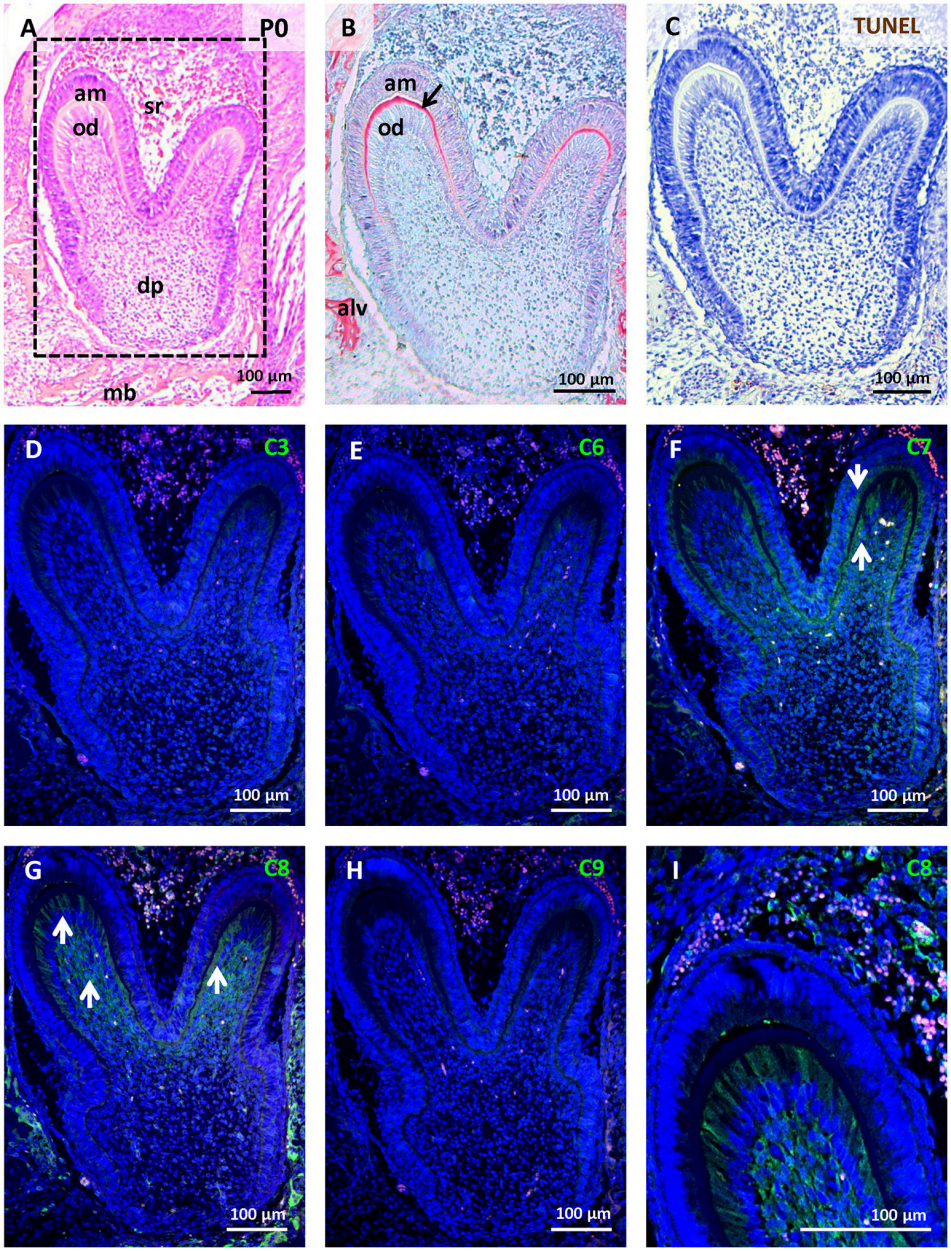
Activation of caspases in the first mandibular molar at P0. Molar morphology (differentiation/matrix secretion) visualised by haematoxylin-eosin staining **(A)**, dentin (black arrow) formation stained by trichrome **(B)**, apoptotic cells labelled by TUNEL assay **(C)**, immunofluorescent detection (green) of caspase-3 **(D)**, caspase-6 **(E)**, caspase-7 **(F)**, caspase-8 **(G,I)**, caspase-9 **(H)**. Nuclei were counterstained by DAPI (blue). Alv (alveolus), am (ameloblasts), dp (dental pulp), mb (mandibular bone), od (odontoblasts), sr (stellate reticulum). Arrows point to positive cells.

Only sporadic apoptotic cells were found in the tooth germ (Figure 4C). Caspase-3 (Figure 4D), caspase-6 (Figure 4E), and caspase-9 (Figure 4H) were not apparent in the tooth at this stage. Activated caspase-7 was present in pre-odontoblasts/odontoblasts, pre-ameloblasts/ameloblasts, and some cells of the dental pulp (Figure 4F). Activation of caspase-7 displayed a gradient with the strongest signal in the coronal part of the tooth germ, thus positively correlating with the gradient of differentiation. Caspase-8 showed a similar pattern to caspase-7, with additional activation in the stellate reticulum (Figures 4G,I). In all of these cell types, activation of caspases-7 and -8 was not linked with TUNEL-positive cells and activation showed cytoplasmic localisation.

### Activation of caspases in mandibular/alveolar bone at P0

**Figure 5:**
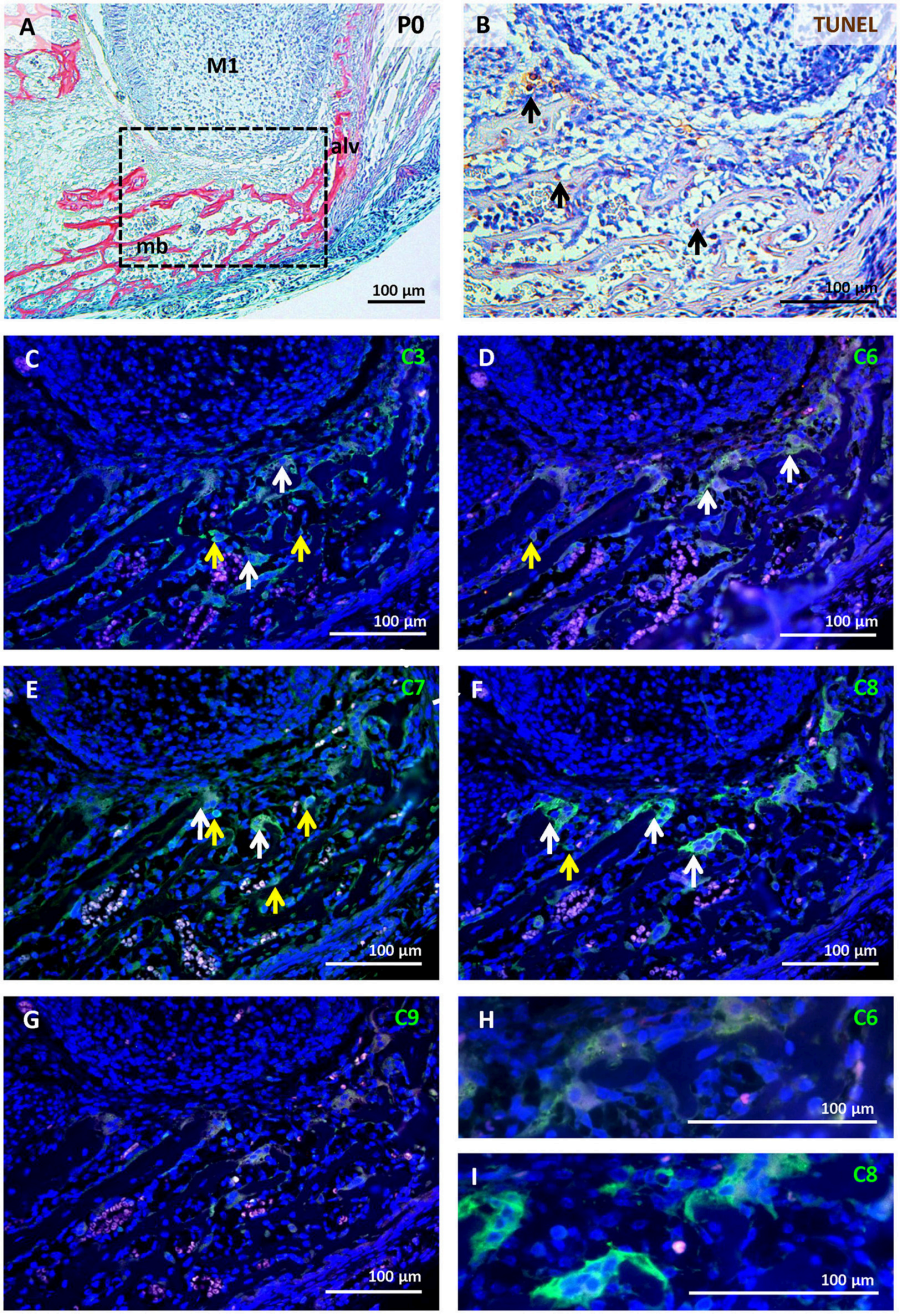
Activation of caspases in the mandibular/alveolar bone at P0. Bone morphology visualised by trichrome staining **(A)**, apoptotic cells (brown) labelled by TUNEL assay **(B)**, immunofluorescent detection (green) of caspase-3 **(C)**, caspase-6 **(D,H)**, caspase-7 **(E)**, caspase-8 **(F,I)**, caspase-9 **(G)**. Nuclei were counterstained by DAPI (blue). Alv (alveolus), M1 (first molar), mb (mandibular bone). White arrows point to positive osteoclasts, yellow arrows point to positive osteoblasts.

A relatively high level of apoptosis was observed in the remodelling bone under the tooth, where the roots will start to develop (Figure 5B). High levels of activated caspases were found in this area. Caspase-3 was activated in few osteoblasts and some osteoclasts, as identified by multinucleated morphology of osteoclasts (Figure 5C). Low activation of caspase-6 was observed in osteoclasts and sporadically in osteoblasts (Figures 5D,H). Caspase-7 activation was apparent in both osteoblasts and osteoclasts (Figure 5E). Caspase-8 was present in some osteoblasts and abundantly activated in osteoclasts (Figures 5F,I). Some of the cells with activated caspases showed apoptotic morphology but the majority did not, and in those cases their expression was cytoplasmic. Very low activation of caspase-9 was observed in osteoclasts at this stage (Figure 5G). The presence of active caspases in different cell types at different stages is summarised in Table 1 and documented in detail in Figures S1, S2. The selection of developmental stages in order to investigate different stages of odontoblast differentiation was based on earlier published data (reviewed in Lisi et al., 2003).

**Table 1:** Summarising table of observed activation of apoptotic caspases.

	**Caspase-3**	**Caspase-6**	**Caspase-7**	**Caspase-8**	**Caspase-9**
Primary enamel knot	^***^	^–^	^***^	^–^	^***^
Enamel epithelium	^–^	^–^	^**^	^*^	^–^
Pre-/ameloblasts	^–^	^–^	^**^	^*^	^–^
Stellate reticulum	^–^	^–^	^–^	^***^	^–^
Dental mesenchyme	^–^	^–^	^–^	^–^	^–^
Pre-/odontoblasts	^–^	^–^	^**^	^**^	^–^
Pre-osteoblasts	^–^	^–^	^–^	^*^	^*^
Osteoblasts (prenatal)	^*^	^*^	^**^	^**^	^–^
Osteoblasts (postnatal)	^*^	^–^	^***^	^*^	^–^
Osteocytes	^*^	^–^	^**^	^*^	^–^
Lining cells	^–^	^–^	^*^	^**^	^–^
Osteoclasts (prenatal)	^*^	^*^	^**^	^***^	^–^
Osteoclasts (postnatal)	^*^	^*^	^***^	^***^	^*^

– negative;

* weak activation;

** medium activation;

*** *abundant activation*.

### Activation of caspases in an osteoblastic cell line

**Figure 6:**
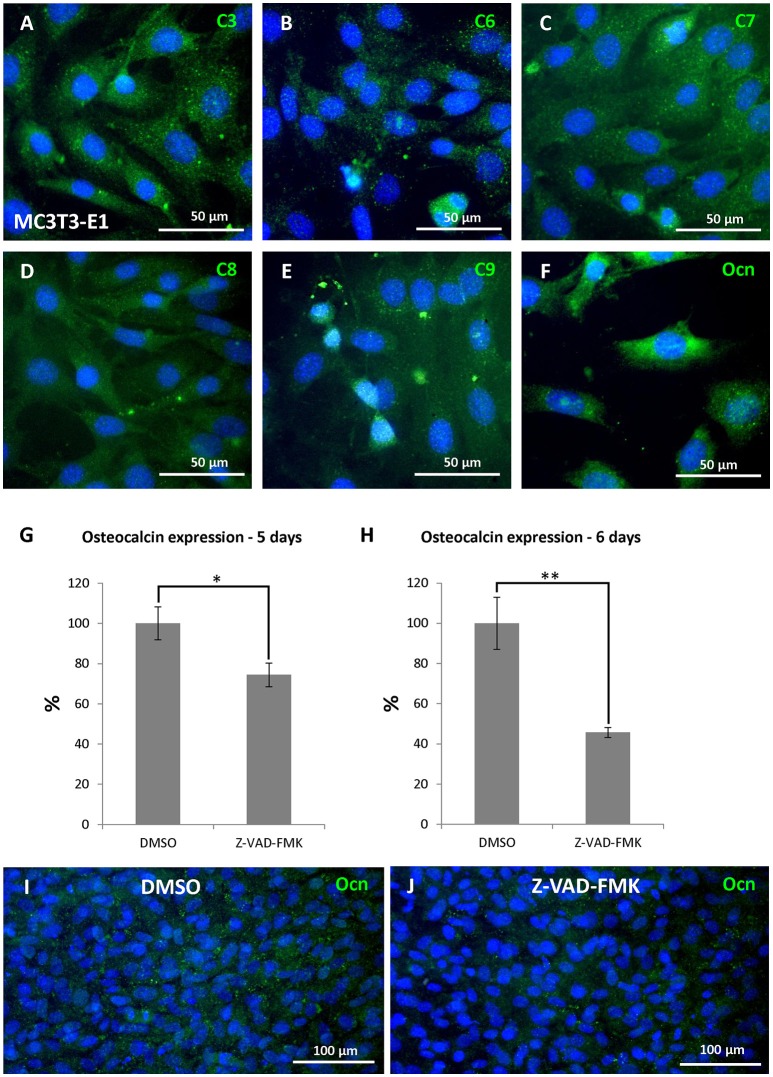
Caspase engagement in differentiation of osteoblastic cells. Activation of caspases in MC3T3-E1 culture after 21 days of osteoblastic differentiation: caspase-3 **(A)**,−6 **(B)**,−7 **(C)**,−8 **(D)**,−9 **(E)**, expression of osteocalcin **(F)**, decrease in osteocalcin expression to 74 % after 5 days of caspase inhibition **(G)**, decrease in osteocalcin expression to 46 % after 6 days of caspase inhibition **(H)**. Expression of osteocalcin detected at the protein level **(J)** compared with control samples **(I)**, showing loss of cytoplasmic osteocalcin. Ocn (osteocalcin). ^*^p ≤ 0.05, ^**^p ≤ 0.01.

The high level of cytoplasmic caspase expression shown in the developing mandibular/alveolar bone was then compared to expression in a bone cell line. For this, the osteoblastic MC3T3-E1 cell line was chosen as this line is derived, like alveolar bone, from cells that form bone via intramembranous ossification. Cells were stimulated using a standard osteogenic medium for 3 weeks to allow differentiation of bone cells (Yazid et al., 2010). After culture, the cells showed activation of all caspases under study (Figures 6A–E) as well as expression of osteocalcin, an important marker of osteoblastic differentiation (Figure 6F). In agreement with the *in vivo* data, predominantly cytoplasmic localisation was observed in all cases.

### Caspase inhibition in an osteoblastic cell line

To study the role of caspases during osteoblast differentiation, a general caspase inhibitor was applied to cultured MC3T3-E1 cells for up to 6 days. The treated cells appeared normal over this culture period, reaching confluence at the same stage as controls. Having established that osteocalcin is expressed at high levels in control cultures, we concentrated on expression of this key gene. A statistically significant decrease in osteocalcin expression (Figures 6G,H) was observed after 5 days of inhibition compared to control, DMSO treated cultures (decrease to 74%, *p* = 0.01203). A more pronounced decrease was noted at 6 days (decrease to 46%, *p* = 0.00263). The loss of osteocalcin RNA was confirmed by loss of osteocalcin protein in cell culture by immunofluorescence (Figures 6I,J).

## Discussion

In this investigation, a comprehensive analysis of pro-apoptotic caspase activation in the developing tooth and bone was provided. The results further highlight important roles for caspases during odontogenesis and osteogenesis, not only in apoptosis but also in non-apoptotic processes and suggest both unique and overlapping functions of the different caspases.

At the late cap stage, the primary enamel knot (PEK) is apoptotically eliminated (Vaahtokari et al., 1996; Jernvall et al., 1998; Shigemura et al., 2001) and osteoclasts are first observed lining the forming alveolar bone, which will gradually encapsulate the tooth (Alfaqeeh et al., 2013). The initiator caspase of the intrinsic pathway, caspase-9, has previously been reported to be essential for apoptosis in the PEK (Setkova et al., 2007), and caspase-9 deficiency was shown to result in reduced apoptosis (Kuida et al., 1998). In contrast, caspase-8 was not observed in this structure, supporting that cell death in this structure is driven via the intrinsic pathway. Of the executioner caspases, caspase-3 activation was detected in apoptotic cells of the molar PEK at this stage (Matalova et al., 2006), and was not observed in the tooth at later stages, suggesting that the role of caspase-3 in the tooth is limited to removal of the PEK. Activated caspase-7 was also present in apoptotic cells of the PEK, but it had a much wider expression in the tooth compared to caspase-3, being expressed in other parts of the dental epithelium at the cap stage and in odontoblasts and ameloblasts at later stages. The more widespread expression of caspase-7, in contrast to caspase-3, supports the previous suggestion that the functions of caspases-3 and -7 are partially distinct and may not act redundantly (Chandler et al., 1998; Walsh et al., 2008; Nakatsumi and Yonehara, 2010). The last of the executioner trio of caspases, caspase-6, had not previously been investigated in odontogenesis and osteogenesis. In our study, activation of caspase-6 did not occur within the tooth germ, and, therefore, does not function in PEK removal. Activation of caspases-3, -7, and -9 was localised to the nucleus of the PEK cells, agreeing with previous suggestions that nuclear localisation is associated with apoptotic roles (Faleiro and Lazebnik, 2000; Fischer et al., 2003; Kamada et al., 2005).

Later in tooth development, caspase-8 and caspase-7 were identified in differentiating/differentiated odontoblasts and ameloblasts as well as cells of dental pulp within the first molar. The expression of activated caspase-8 displayed a gradient, copying the differentiation pattern of these cells (Schmitt and Ruch, 2000). Expression of both caspases was cytoplasmic and did not overlap with apoptosis, suggesting non-apoptotic roles during development. The expression of caspases-7 and -8 in epithelial-derived tissue suggests a novel role of these two caspases in ameloblasts development.

In the bone at the prenatal stage and later in the perinatal period, caspase-3 was localised mostly in non-apoptotic osteoclasts and a few osteoblasts, agreeing with previous detection in human tissue; however, apoptotic/non-apoptotic correlation was not performed (Krajewska et al., 1997). Active caspase-7, again, had a wider expression pattern compared to caspase-3, and was identified in non-apoptotic osteoblasts, osteocytes, lining cells, and osteoclasts. Caspase-6 was also apparent in osteoblasts and osteoclasts, but decreased in perinatal osteoblasts. This change could be explained by declining age-specific activation observed in other tissues (Godefroy et al., 2013) and suggests a distinct role of caspase-6 in developing osteoblasts. Notably, all three executioner caspases were localised in the cytoplasm of non-apoptotic cells in the bone, in contrast to their nuclear expression in the PEK. Subcellular localisation of pro-apoptotic caspases was proposed as one option for the diversification between apoptotic and non-apoptotic functions (Faleiro and Lazebnik, 2000; Fischer et al., 2003; Kamada et al., 2005).

Caspase-9, had not previously been studied in the intramembranous bone *in vivo*. Nevertheless, caspase-9 was demonstrated *in vitro* to be activated in the case of BMP-2 mediated apoptosis in primary human calvaria osteoblasts and in immortalised human calvaria osteoblasts (Haÿ et al., 2001). Localisation of caspase-9 in the mandibular/alveolar bone was associated with pre-osteoblastic differentiation and with osteoclasts.

Activation of caspase-8 was apparent in some apoptotic cells, which is in accordance with previously published findings showing caspase-8 activated in apoptotic osteoblasts or osteocytes *in vitro* (Kogianni et al., 2004; Goga et al., 2006; Aoyama et al., 2012; Thaler et al., 2016). Nevertheless, in the mandibular bone activation of caspase-8 was particularly associated with non-apoptotic osteoblastic cells (pre-osteoblasts, osteoblasts, osteocytes, and lining cells) and osteoclasts. The finding in osteoblastic cells is supported by earlier observations *in vitro*, where activation of caspase-8 was associated with osteoblast differentiation (Mogi and Togari, 2003). Regarding osteoclasts, these cells are derived from the monocyte macrophage lineage and, thus, appearance of active caspase-8 may be related to its role in monocyte differentiation (Kang et al., 2004). Non-apoptotic function of caspase-8 in myeloid cells was suggested to act via modification of nuclear factor-kappa B signalling (Solier et al., 2017), which might also be the case in osteoclasts. The mechanism of non-apoptotic stimulation of caspase-8 has not yet been clarified, but one of the proposals is functional divergence based on altered substrate specificity (Pop et al., 2011).

Substrate specificity of caspases determined by post-translational modifications and interactions with other proteins is often suggested as a mechanism for non-apoptotic function of pro-apoptotic caspases (Zermati et al., 2001; Sordet et al., 2002; Nhan et al., 2006; Shalini et al., 2015). Caspases, in general, target hundreds of substrates including not only direct regulators of apoptosis but also molecules participating in cell adhesion, cytoskeleton formation, nuclear structural proteins, ER and Golgi associated proteins, cell cycle regulators, proteins related to DNA synthesis, cleavage and repair, transcription factors, molecules associated with RNA synthesis and protein translation as well as cytokines, membrane receptors, and others (Fischer et al., 2003; Shalini et al., 2015). Determination of the exact pathways associated to the non-apoptotic activity of caspases in odontogenesis and related osteogenesis, such as modulation of osteocalcin expression, remains to be clarified.

Our investigation provided a comprehensive analysis of pro-apoptotic caspase activation in different cell types related to their apoptotic and non-apoptotic status during odontogenesis and intramembranous osteogenesis. The activation patterns of caspase-8 and caspase-6 in developing teeth and alveolar bone are reported here for the first time as well as the non-apoptotic presence of caspase-9. Additionally, the significant and not yet known impact of caspase inhibition on osteocalcin expression has been demonstrated.

## Author contributions

ES: Preliminary data, histological staining, RNA isolation, cDNA transcription, manuscript construction; BV: Immunofluorescence, immunocytochemistry, cell cultures, qPCR, manuscript preparation; AT: Discussion of experiments, manuscript preparation; EM: Preliminary analysis, manuscript preparation, head of the project.

### Conflict of interest statement

The authors declare that the research was conducted in the absence of any commercial or financial relationships that could be construed as a potential conflict of interest.

## Supplementary material

Figure S1 Activation of caspases in osteoblastic cells detected in mandibular/alveolar bone. Morphology (haematoxylin-eosin) of mandibular bone at E13 **(A)**, activation of caspase-8 in pre-osteoblastic cells at E13 **(B)**, activation of caspase-9 in pre-osteoblastic/osteoblastic cells at E13 **(C)**, morphology (haematoxylin-eosin) of mandibular/alveolar bone at E15 **(D)**, activation of caspase-6 in osteoblasts at E15 **(E)**, correlated with localisation of osteocalcin-positive cells at E15 **(F)**, activation of caspase-7 in osteoblasts at E15 **(G)**, correlated with osteocalcin-positive cells at E15 **(H)**, activation of caspase-7 in lining cells at E15 **(I)**, correlated with osteocalcin-negative cells at E15 **(J)**, activation of caspase-8 in osteoblasts/lining cells at E15 **(K)**, correlated with osteocalcin-positive/osteocalcin-negative cells at E15 **(L)**, morphology (haematoxylin-eosin) of mandibular/alveolar bone at P22 **(M)**, activation of caspase-7 in matrix incorporated osteocytes at P22 **(N)**, activation of caspase-8 in matrix incorporated osteocytes at P22 **(O)**. Mc (Meckel's cartilage), mb (mandibular bone). White arrows point to positive cells, yellow arrows point to negative.

Figure S2 Activation of caspases in odontoblastic cells detected in the first molar of mandible. Morphology (haematoxylin-eosin) of the first molar at stage E18 **(A)** and pre-dentin detection (arrow) by trichrome **(A**_1_**)**, activation of caspase-7 in pre-odontoblasts—future crown segment (pronounced differentiation) at E18 **(B)**, activation of caspase-7 in pre-odontoblasts—future root segment (retarded differentiation) at E18 **(C)**, activation of caspase-8 in pre-odontoblasts—future crown segment at E18 **(D)**, activation of caspase-8 in pre-odontoblasts—future root segment at E18 **(E)**, morphology (haematoxylin-eosin) of the first molar at stage P22 **(F)** activation of caspase-7 in odontoblasts—crown segment (pronounced differentiation) at P22 **(G)**, activation of caspase-7 in odontoblasts—root segment (retarded differentiation) at P22 **(H)**, activation of caspase-8 in odontoblasts—crown segment at P22 **(I)**, activation of caspase-8 in odontoblasts—root segment at P22 **(J)**. Ab, alveolar bone; am, ameloblasts; dp, dental pulp; mb, mandibular bone; od, odontoblasts. Arrows point to positive cells.

## Abbreviations

E: embryonic day
P: postnatal day
PEK: primary enamel knot.
