# Supplementary figures and images for: Activation of Pro-apoptotic Caspases in Non-apoptotic Cells During Odontogenesis and Related Osteogenesis
Source: Front Physiol. 2018 Mar 7;9:174. doi: 10.3389/fphys.2018.00174 (PMC5845891; doi:10.3389/fphys.2018.00174)

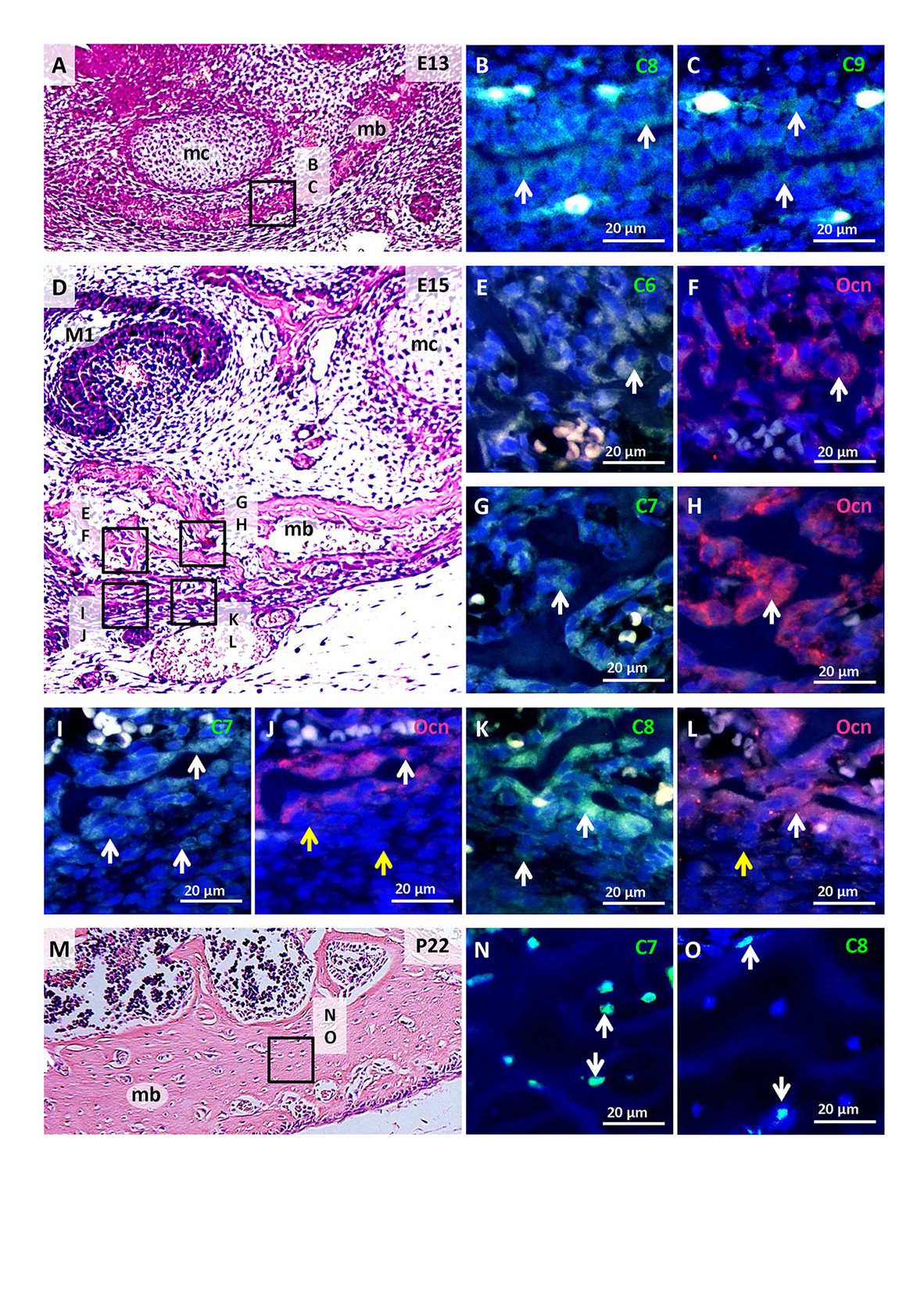

Supplement: Figure S1 — Activation of caspases in osteoblastic cells detected in mandibular/alveolar bone. Morphology (haematoxylin-eosin) of mandibular bone at E13 (A), activation of caspase-8 in pre-osteoblastic cells at E13 (B), activation of caspase-9 in pre-osteoblastic/osteoblastic cells at E13 (C), morphology (haematoxylin-eosin) of mandibular/alveolar bone at E15 (D), activation of caspase-6 in osteoblasts at E15 (E), correlated with localisation of osteocalcin-positive cells at E15 (F), activation of caspase-7 in osteoblasts at E15 (G), correlated with osteocalcin-positive cells at E15 (H), activation of caspase-7 in lining cells at E15 (I), correlated with osteocalcin-negative cells at E15 (J), activation of caspase-8 in osteoblasts/lining cells at E15 (K), correlated with osteocalcin-positive/osteocalcin-negative cells at E15 (L), morphology (haematoxylin-eosin) of mandibular/alveolar bone at P22 (M), activation of caspase-7 in matrix incorporated osteocytes at P22 (N), activation of caspase-8 in matrix incorporated osteocytes at P22 (O). Mc (Meckel's cartilage), mb (mandibular bone). White arrows point to positive cells, yellow arrows point to negative. [file Image1.TIF]

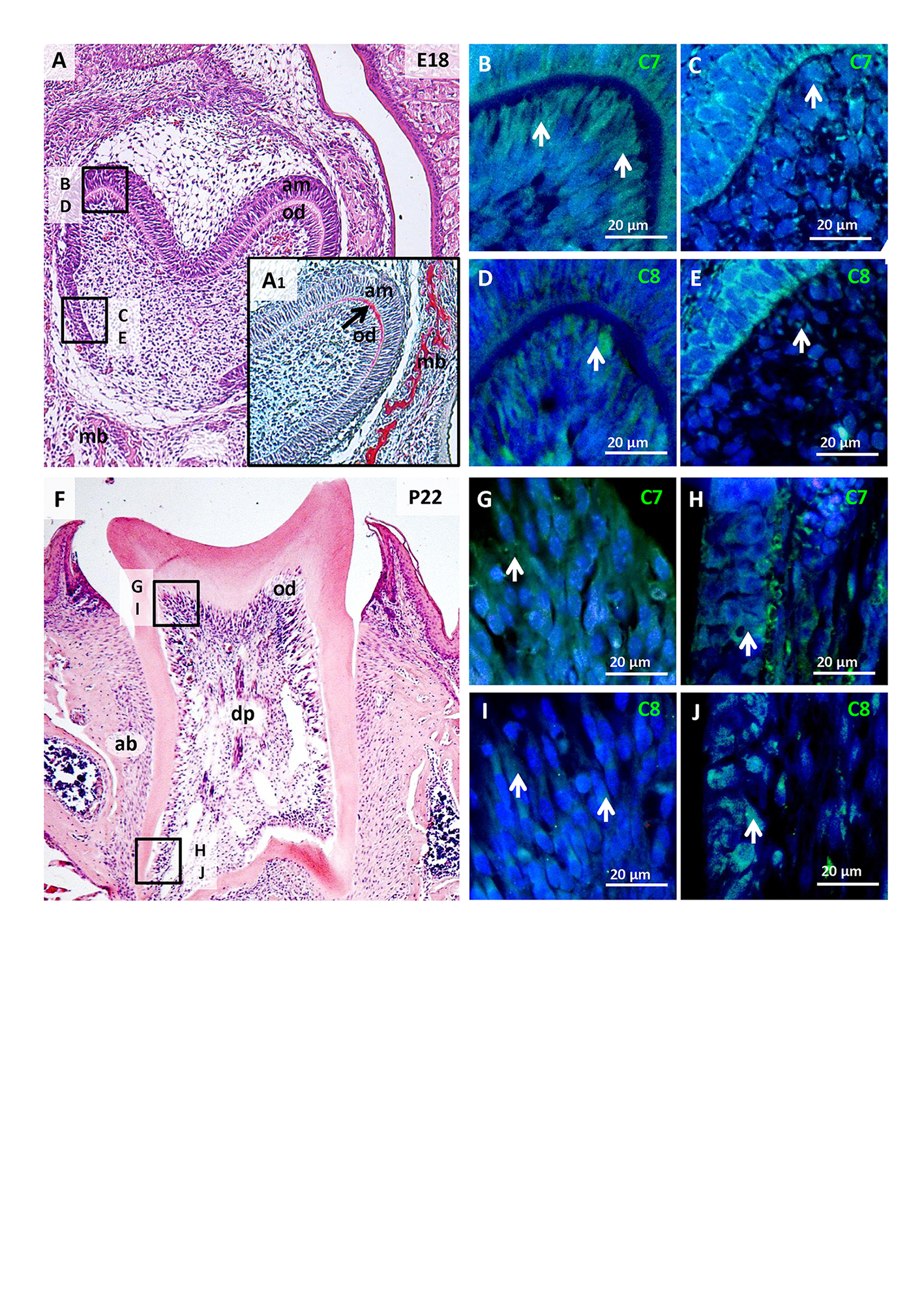

Supplement: Figure S2 — Activation of caspases in odontoblastic cells detected in the first molar of mandible. Morphology (haematoxylin-eosin) of the first molar at stage E18 (A) and pre-dentin detection (arrow) by trichrome (A1), activation of caspase-7 in pre-odontoblasts—future crown segment (pronounced differentiation) at E18 (B), activation of caspase-7 in pre-odontoblasts—future root segment (retarded differentiation) at E18 (C), activation of caspase-8 in pre-odontoblasts—future crown segment at E18 (D), activation of caspase-8 in pre-odontoblasts—future root segment at E18 (E), morphology (haematoxylin-eosin) of the first molar at stage P22 (F) activation of caspase-7 in odontoblasts—crown segment (pronounced differentiation) at P22 (G), activation of caspase-7 in odontoblasts—root segment (retarded differentiation) at P22 (H), activation of caspase-8 in odontoblasts—crown segment at P22 (I), activation of caspase-8 in odontoblasts—root segment at P22 (J). Ab, alveolar bone; am, ameloblasts; dp, dental pulp; mb, mandibular bone; od, odontoblasts. Arrows point to positive cells. [file Image2.TIF]
